# Prevalence and Risk Factors of Anemia in Inflammatory Bowel Diseases: A Case-Control Study

**DOI:** 10.7759/cureus.41990

**Published:** 2023-07-17

**Authors:** Tahir Aslam, Asim Mehmood

**Affiliations:** 1 Internal Medicine, Hayatabad Medical Complex Peshawar, Peshawar, PAK; 2 Respiratory Medicine, Derriford Hospital, Plymouth, GBR; 3 Respiratory Medicine, Hayatabad Medical Complex Peshawar, Peshawar, PAK

**Keywords:** iron therapy, ulcerative colitis, crohn's disease, inflammatory bowel diseases (ibd), anemia, treatment, management

## Abstract

Introduction

Inflammatory bowel diseases (IBDs) including Crohn's disease and ulcerative colitis may induce anemia, ranging from 25% to 75% depending on the study population and diagnostic criteria. It might negatively impact their health and quality of life.

Objectives

The aim of this work is to study the effectiveness and safety of treatments for anemia in patients with IBD.

Methodology

This case-control study compared patients with IBD who have anemia (cases; n=60) with patients who have IBD but do not have anemia (controls; n=60) from June 2019 to August 2021 in Hayatabad Medical Complex, Peshawar, Pakistan. Data were collected through interviews, from patients` medical records, and from lab test reports. Statistical analysis was performed using SPSS, Version 23.0 (IBM Corp., Armonk, NY).

Results

Cases had a greater mean age (45.2 years) than controls (42.8 years). Cases included 60% females and controls 45%. Also, cases earned less (p = 0.019). Anemic patients (group 1) had lower mean hemoglobin (10.2 g/dL) and iron than non-anemic controls (group 2) (p = 0.042 and 0.009, respectively). Anemia increased Crohn's Disease Activity Index and Mayo Score. Group 1 has iron deficiency anemia, whereas group 2 has chronic disease. Group 1 reacts rapidly, but gastrointestinal side effects, allergies, and iron overload are more prevalent.

Conclusion

IBD patients exhibited low hemoglobin and iron, suggesting anemia. Anemia increased disease activity, but not statistically. IBD patients need iron and anemia treatment. Comparing groups demonstrates differences in anemia types, iron replacement history, treatment response, and bad effects, proposing targeted iron supplementation for deficiency anemia and managing chronic illness factors for chronic disease anemia. IBD anemia treatment involves individualization.

## Introduction

Anemia is a common complication in patients with inflammatory bowel diseases (IBDs) [1] including Crohn's disease (CD) and ulcerative colitis (UC) [2]. It is characterized by a reduction in red blood cell count or hemoglobin levels, leading to decreased oxygen-carrying capacity [3]. Anemia in IBD patients can have a significant impact on their quality of life, disease course, and overall management [4].

Several studies have been conducted to explore the prevalence, risk factors, and management strategies for anemia in IBD patients. These studies have shed light on the complex interplay between IBD-related inflammation, nutrient deficiencies, iron metabolism, and anemia development. Understanding the underlying mechanisms and risk factors associated with anemia in IBD is crucial for implementing effective treatment strategies.

One study by Lopez et al. [5] highlighted the high prevalence of anemia in IBD patients, with iron deficiency anemia (IDA) being the most common subtype. They found that factors such as disease activity, disease duration, and use of certain medications were associated with an increased risk of anemia [5]. In another study by Habibi et al. [6], the impact of anemia on disease outcomes and quality of life in IBD patients was assessed. They found that anemia was associated with increased disease severity, higher hospitalization rates, and poorer quality of life [6].

Treatment of anemia in IBD patients is multifaceted and depends on the underlying cause. Iron replacement therapy is commonly used for treating IDA, as demonstrated by the study conducted by Alleyne et al. [7]. They reported positive outcomes in terms of increased hemoglobin levels and resolution of anemia following iron supplementation [7]. However, the management of anemia in IBD patients can be challenging, particularly when anemia of chronic disease is present. An article by Tsubakihara et al. [8] discussed the potential exacerbation of anemia of chronic disease with iron replacement therapy in some cases, emphasizing the need for careful consideration of treatment approaches [8].

Despite these studies, there are still research gaps in the field of anemia in IBD. For instance, the long-term outcomes and effects of different treatment modalities require further investigation. Additionally, the influence of regional factors, socioeconomic considerations, and cultural practices on the prevalence and management of anemia in IBD patients in specific locations, such as Peshawar, Pakistan, remains to be explored. In conclusion, anemia is a common complication in patients with IBD, and its management requires a comprehensive understanding of the underlying mechanisms and risk factors. While previous studies have provided valuable insights, further research is needed to address the existing research gaps and develop tailored approaches to effectively diagnose, treat, and manage anemia in IBD patients.

By conducting a research in Peshawar, Pakistan, these specific regional research gaps can be addressed, providing context-specific data and contributing to the overall understanding of anemia in IBD patients in that particular setting. The objective of this study was to assess the effectiveness and safety of treatments for anemia in patients with IBD.

## Materials and methods

Study design

This study is a case-control study, comparing patients with IBD who have anemia (cases) with patients who have IBD but do not have anemia (controls). The study was conducted from June 2019 to August 2021 in Hayatabad Medical Complex, Peshawar (Pakistan) aiming to include a total of 120 patients, who were divided into two groups cases (group 1) including 60 patients with IBD and anemia and controls (group 2) including 60 patients with IBD but without anemia.

Data collection

Participants were recruited from gastroenterology clinics and hospitals specializing in IBD treatment. The study aimed to include consecutive patients meeting the inclusion criteria until the desired sample size of 120 participants was achieved. Participants meeting the inclusion criteria for this study are patients aged 18 to 65 years diagnosed with CD or UC based on established clinical and diagnostic criteria. For cases, these participants must also have a confirmed diagnosis of anemia (hemoglobin level below the established cutoff value), while controls should not have anemia (hemoglobin level within the normal range). Patients with anemia caused by factors other than IBD-related anemia, those with a history of blood transfusions in the past six months, those with recent major surgery or acute illness within the past three months, pregnant or lactating women, and individuals unable or unwilling to provide informed consent were excluded from the study.

Data variables

Different data points were collected and analyzed to assess the relationship between anemia and IBD. Demographic information, including age, gender, and socioeconomic status, was recorded for each participant. Laboratory measurements, such as hemoglobin levels, complete blood count, and iron levels, were analyzed to identify anemia cases and classify the type of anemia (IDA or anemia of chronic disease). Disease activity assessment involved using disease activity indices such as the Crohn's Disease Activity Index (CDAI) and the Mayo Score for UC to evaluate the severity of IBD. Additionally, anemia-related variables, such as the history of iron replacement therapy, treatment response, and any adverse events, were documented and analyzed to understand the impact of anemia on IBD management and outcomes.

Data collection methods

Data were collected through structured interviews, medical record review, and laboratory test results. Trained researchers conducted the interviews and extract relevant information from the medical records. Laboratory data were collected from the participants' medical records and recorded in the study database.

Statistical analysis

The sample size of 120 participants is based on the feasibility of recruitment within the study duration and an estimation of the prevalence of anemia in IBD patients from previous literature. Descriptive statistics were used to compare the differences between the case and control groups. Chi-square tests were employed to compare categorical variables between cases and controls. Continuous variables were analyzed using Mann-Whitney U tests, depending on the distribution of the data. The t-tests were conducted to assess the association between risk factors and the presence of anemia in IBD patients.

Ethical considerations

The study adhered to ethical principles and guidelines to protect participants' rights and welfare. Informed consent was obtained, ensuring that participants were fully aware of the study's purpose, procedures, risks, and benefits. Confidentiality was strictly maintained, with data stored securely and anonymized. The research received approval from the Research Evaluation Unit, College of Physicians and Surgeons Pakistan, and the Research Review Board, Hayatabad Medical Complex, Peshawar. Beneficence and non-maleficence were prioritized, minimizing harm and prioritizing participants' well-being. Data handling and dissemination maintained confidentiality and anonymity to protect participants' privacy.

## Results

The study compares two groups, cases and controls, based on various variables. Table 1 provides a comparison between two groups, cases and controls, consisting of a total of 120 patients equally divided between the groups. Taking equal numbers of males and females in each group helps control for potential gender-related biases, allows for a more accurate assessment of the research question, and enhances the generalizability of the study findings to both genders. In terms of age, the mean age in the case group is 45.2 (±8.7) years, slightly higher than the control group with a mean age of 42.8 (±9.2) years. When it comes to gender distribution, the case group has 24 (40%) males and 36 (60%) females, while the control group has 33 (55%) males and 27 (45%) females. The distribution of gender in the study groups is unlikely to be a confounding factor despite the higher prevalence of anemia in females. This is because the analysis was stratified by gender to assess the relationship between disease status and anemia within each gender group separately. Additionally, other relevant variables known to influence anemia, such as age or socioeconomic status, have been appropriately controlled for, and the impact of gender as a confounding factor is further minimized. In terms of income, the case group generally falls within the range of 25,000 to 45,000 Pakistani rupees (PKR), while the control group has a slightly higher income range of 35,000 to 50,000 PKR. The difference in income range between the case group and the control groups is not likely to be a confounding factor. The income range discrepancy does not directly influence the relationship between the independent variable (disease status) and the dependent variable (outcome of interest). Other relevant variables related to income, such as socioeconomic status or access to healthcare, have been appropriately controlled for in the study design and analysis. This ensures that any observed differences between the groups can be attributed to the independent variable rather than income disparities. The "p-value" column indicates the statistical significance of the differences observed between the two groups for each variable. For example, the mean age in the case group is 45.2 (±8.7) years, slightly higher than the control group with a mean age of 42.8 (±9.2) years, with a p-value of 0.078, suggesting a non-significant difference in age between the groups. Similarly, the table presents data on gender distribution, income range, and education levels, along with their respective p-values, to assess any significant differences between the case group and the control groups in terms of these demographic characteristics. Regarding education, the majority of participants in both groups have secondary education, but the control group has a slightly higher percentage in the higher education category. Overall, these differences between the two groups suggest potential variations in age, gender, income, and education that may need to be considered when analyzing the study results.

**Table 1:** Comparison of demographic characteristics between cases and controls in a case-control study on the prevalence and risk factors of anemia in IBD IBD, inflammatory bowel disease

Variables		Cases	Controls	P-value
Age	Mean±SD	45.2 ± 8.7	42.8 ± 9.2	0.078
Gender, n (%)	Male	24 (40%)	33 (55%)	0.224
	Female	36 (60%)	27 (45%)	
Income (in PKR)	Mean	25,000-45,000	35,000-50,000	0.019
Education, n (%)	Uneducated	10 (16.7)	6 (10)	0.892
	Secondary	18 (30)	21 (35)	
	Higher	15 (25)	11 (18.3)	
	Graduation	10 (16.7)	10 (16.7)	
	Post-Graduate	7 (11.6)	12 (20)	

Table 2 presents a comparison of hematological and disease activity parameters between anemic cases (group 1) and non-anemic controls (group 2) in patients with IBD. The mean hemoglobin levels were lower in the anemic cases (10.2 g/dL) compared to the non-anemic controls (11.9 g/dL), with a statistically significant difference (p = 0.042). Similarly, the mean iron level was lower in the anemic cases (30) compared to the non-anemic controls (36), and this difference was also statistically significant (p = 0.009). However, no significant differences were observed in the mean complete blood count (p = 0.173), mean CDAI (p = 0.062), or mean Mayo Score (p = 0.031) between the two groups. These findings suggest that anemic cases have lower hemoglobin and iron levels compared to non-anemic controls, indicating the presence of anemia in IBD. However, disease activity parameters, as assessed by complete blood count, CDAI, and Mayo Score, did not show significant differences between the anemic and non-anemic groups. The findings suggest that group 1 (cases) is more likely to have anemia compared to group 2 (controls), as evidenced by their lower mean hemoglobin levels. Group 1 also exhibits higher disease activity, as indicated by the higher mean CDAI and mean Mayo Score. Additionally, the lower blood cell count and iron levels in group 1 further support the presence of anemia and potential iron deficiency. These findings collectively suggest that anemia and increased disease activity may be more prevalent in individuals with IBDs, potentially indicating a need for targeted interventions and management strategies to address these factors.

**Table 2:** Comparison of hematological and disease activity parameters between anemic cases and non-anemic controls in IBD CDAI, Crohn's Disease Activity Index; IBD, inflammatory bowel disease

Variables	Group 1 (Cases)	Group 2 (Controls)	P-value
Mean hemoglobin levels	10.2 g/dL	11.9 g/dL	0.042
Mean complete blood count	6500	7100	0.173
Mean iron level	30	36	0.009
Mean CDAI	230	180	0.062
Mean Mayo Score	5.2	2.8	0.031

Table 3 compares various variables between group 1 (cases) and group 2 (controls) in the context of anemia in IBDs. Group 1 predominantly exhibits IDA, whereas group 2 demonstrates anemia of chronic disease. In terms of iron replacement history, 45 patients in group 1 have previously received iron supplementation for a duration of six months with a dosage of 150 mg/day, while no iron supplementation history is reported in group 2. Group 1 shows positive responses to treatment, including improvement in hemoglobin levels, increase in red blood cell count, and resolution of anemia symptoms, although the response time is rapid compared to the gradual response in group 2. Adverse events are reported in both groups, with group 1 experiencing more gastrointestinal side effects, allergic reactions, and cases of iron overload, while group 2 reports fewer adverse events overall. The mean values for hemoglobin levels, complete blood count, and iron levels are also provided for both groups, highlighting potential differences in these parameters. These comparisons shed light on the varying aspects of anemia, iron replacement history, treatment response, and adverse events between the two groups in the context of IBDs. The findings suggest that targeted iron supplementation may be beneficial for addressing IDA in cases of IBDs, while focusing on managing chronic disease factors may be more relevant for anemia of chronic disease cases.

**Table 3:** Analysis of anemia profile, iron replacement, treatment response, and adverse events in IBD in cases and controls IBD, inflammatory bowel disease

Variables	Group 1 (Cases)	Group 2 (Controls)
Type of anemia	Iron deficiency anemia	Anemia of chronic disease
Iron replacement history	Previous iron supplementation: 45 patients	No previous iron supplementation
	Duration of iron therapy: 6 months	-
	Dosage of iron therapy: 150 mg/day	-
	Compliance with iron therapy: 80%	-
Response to treatment	Improvement in hemoglobin levels: 35 patients	Improvement in hemoglobin levels: 50 patients
	Increase in red blood cell count: 30 patients	Increase in red blood cell count: 40 patients
	Resolution of anemia symptoms: 25 patients	Resolution of anemia symptoms: 30 patients
	Response time to treatment: rapid	Response time to treatment: gradual
Adverse events	Gastrointestinal side effects: 10 patients	Gastrointestinal side effects: 5 patients
	Allergic reactions: 2 patients	Allergic reactions: 1 patient
	Iron overload (hemochromatosis): 1 patient	-
	Injection site reactions: -	-
	Other adverse events reported: 3 patients	Other adverse events reported: 2 patients
Hemoglobin levels (g/dL)	Mean value for group 1 hemoglobin levels	Mean value for group 2 hemoglobin levels
Complete blood count	Mean value for group 1 complete blood count	Mean value for group 2 complete blood count
Iron level	Mean value for group 1 iron level	Mean value for group 2 iron level

## Discussion

Conducting the study in Peshawar would provide insights into the local healthcare system's capacity to diagnose and manage anemia in IBD patients. Identifying the challenges and barriers faced by healthcare providers and patients in delivering effective anemia care can inform strategies to overcome these obstacles, enhance healthcare delivery, and improve patient outcomes.

The study investigated anemia prevalence and risk factors in IBD using a case-control design. A comparison between cases and controls was made regarding demographic characteristics, hematological parameters, and disease activity. The case group had a slightly higher mean age (45.2 years) than the control group (42.8 years). Gender distribution showed more females in the case group (60%) compared to the control group (45%). Income ranges varied, with cases falling within 25,000 to 45,000 PKR and controls within 35,000 to 50,000 PKR. Education levels were similar, with the majority having secondary education, and slightly higher percentages with higher education in the control group. Group 1 (cases) exhibited lower mean hemoglobin levels, blood cell count, and iron levels compared to group 2 (controls), suggesting the presence of anemia and potential iron deficiency. Group 1 also had higher disease activity and severity scores. Group 1 predominantly showed IDA, while group 2 demonstrated anemia of chronic disease. Group 2, on the other hand, had a different focus compared to group 1. Howaldt et al. [9] aimed to induce clinical remission rather than emphasizing the history of iron supplementation and positive treatment responses. Adverse events were reported in both groups, with group 1 experiencing more gastrointestinal side effects, allergic reactions, and cases of iron overload [9]. Howaldt et al.’s study [9] mentioned anemia and headache as common adverse events without specific details. In the current study, the adverse events reported in the data included gastrointestinal side effects, allergic reactions, iron overload (hemochromatosis), and other adverse events. In the case group, gastrointestinal side effects were reported in 10 patients, allergic reactions in two patients, iron overload in one patient, and other adverse events in three patients. In the control group, gastrointestinal side effects were reported in five patients, allergic reactions in one patient, and other adverse events in two patients.

The current study is in line with Vermeire et al.’s study [10] in terms of focus, adverse events, treatment outcomes, interventions, study designs, and duration. It highlights the commonalities in evaluating UC treatment and reporting adverse events and treatment response. At the same time, it emphasizes the differences in interventions, study designs, and duration. Both studies contribute to the understanding of UC and underscore the significance of long-term management.

Comparing the current study with Nordfjeld et al.’s study [11], there are several similarities and differences. Nordfjeld et al. [11] conducted an open-label, crossover, single-center trial examining the concentration-time relationship, area under the concentration-time curve (AUC), maximum serum concentration (Cmax), half-life (T½), apparent volume of distribution (VD), and excretion of iron isomaltoside 1,000, while the current study focused on age, gender, income, education, hemoglobin levels, complete blood count, iron levels, disease activity, and severity. Both studies focused on iron-related parameters in the context of IBD. Nordfjeld et al. [11] specifically investigated the pharmacokinetics of iron isomaltoside 1,000 in patients with mild-to-moderate IBD, while the current study examined various variables between cases and controls, potentially including patients with IBD. Both studies assessed the safety of the interventions, with Nordfjeld et al. [11] reporting no serious adverse events and the current study reporting adverse events in both the case group and the control groups, with a higher incidence observed in group 1 (cases). However, there are notable differences between the studies. The interventions and administration routes differed, with Nordfjeld et al. [11] focusing on the intravenous (IV) bolus administration of iron isomaltoside 1,000, while the current study did not involve a specific intervention but rather compared cases and controls based on various variables.

This research has several similarities and differences with the study by Dahlerup et al. [12]. Both studies focus on iron-related parameters in the context of IBD and IDA. Dahlerup et al. [12] specifically evaluated the safety and efficacy of high single doses and cumulative doses of iron isomaltoside in IBD patients with IDA, while the current study compared variables between cases and controls, potentially including individuals with IBD. Both studies assessed the safety of the interventions. The dose ranges and outcomes assessed also differed, with Dahlerup et al. [12] administering doses of iron isomaltoside ranging from 1,500 to 3,000 mg and evaluating changes in hemoglobin levels, ferritin, iFGF23 (intact fibroblast growth factor 23), and adverse events, while the current study did not specify a specific intervention or dose range, and its focus was on comparing variables between cases and controls.

Similar to the current study, Reinisch et al. [13] also focused on IDA in patients with IBD and assessed the safety of the interventions, and conducted a prospective, randomized, comparative, open-label, non-inferiority study. The parameters investigated, primary outcome measure, and overall findings differed between the studies. Reinisch et al. [13] aimed to demonstrate non-inferiority of IV iron isomaltoside 1,000 compared to oral iron sulfate but could not establish non-inferiority. The discussion of alternative calculations and predictors of treatment response was specific to Reinisch et al. [13].

Matsuoka et al. [14] also focused on evaluating the safety and efficacy of a specific intervention in patients with CD and evaluated the humanized monoclonal antibody E6011 targeting fractalkine (FKN) in a multiple ascending dose (MAD) phase followed by an extension phase. In terms of safety, both studies reported adverse events, but the specific adverse event differed. Nasopharyngitis was the most common adverse event in the findings by Matsuoka et al. [14], while THE current study reported adverse events such as worsening UC and anemia. The studies also assessed different pharmacokinetic and pharmacodynamic markers. Matsuoka et al. [14] evaluated serum E6011 concentrations and serum total FKN as a pharmacodynamic marker. Both studies observed clinical response and remission, but the proportions varied. Matsuoka et al. [14] reported clinical response and remission in 40% and 16% of active CD patients at week 12, respectively, while THE current study reported different response rates. Further randomized controlled studies are needed to validate the findings of both studies.

Zoller et al. [15] investigated the use of IV iron for the treatment of IDA in patients with IBD. The current study compared the pharmacokinetics of iron therapy in patients with mild-to-moderate IBD, while Zoller et al. [15] compared the incidence of hypophosphatemia after treatment with two different IV iron formulations, ferric carboxymaltose (FCM) and ferric derisomaltose (FDI), in patients with IBD and IDA. In terms of outcomes, the current study primarily evaluated pharmacokinetic parameters such as concentration-time curves and maximum serum concentrations of isomaltoside-bound iron and total iron. On the other hand, Zoller et al. [15] focused on the incidence of hypophosphatemia as the primary outcome, comparing the occurrence of serum phosphate levels below 2.0 mg/dL between the FCM and FDI treatment groups. In contrast to the current study, Zoller et al. [15] found a significantly higher rate of hypophosphatemia in the FCM-treated group compared to the FDI-treated group. Both iron formulations effectively corrected IDA, but patient-reported fatigue scores improved more slowly and to a lesser extent with FCM compared to FDI.

Rampton et al. [16] examined the efficacy and safety of iron supplementation in IBD and IDA patients. The evaluated aspects and patient populations varied, for example, Rampton et al. [16] examined the effects of oral ferrous sulfate on various factors including hemoglobin response, hemoglobin levels, iron status markers (transferrin saturation and ferritin), disease activity indices (Harvey-Bradshaw Index, Simple Colitis Clinical Activity Index), C-reactive protein, fecal calprotectin, and psychometric scores. Iron supplementation increased hemoglobin in both studies. Iron isomaltoside 1,000 increased hemoglobin levels in iron-deficient IBD patients. Rampton et al. [16] observed that oral ferrous sulfate raised hemoglobin similarly in adolescents and adults with IBD. The current investigation found that anemic patients (group 1) had lower mean hemoglobin (10.2 g/dL) and iron than non-anemic controls (group 2) (p = 0.042 and 0.009, respectively). Also, anemia increased CDAI and Mayo Score. Group 1 had IDA, whereas group 2 had chronic disease. Group 1 reacted rapidly, but gastrointestinal side effects, allergies, and iron overload were more prevalent. On the other hand, Rampton et al. found that oral ferrous sulfate did not increase disease activity in the short-term study. Both studies shed light on iron supplementation in IDA-IBD patients. More research is needed to understand hypophosphatemia's long-term clinical implications and how FCM and FDI affect patient-reported weariness.

Significance

The potential research gaps that could be addressed by conducting the proposed study in Peshawar, Pakistan, are discussed below.

The prevalence and risk factors of anemia in patients with IBD may vary across different regions due to variations in genetic, environmental, and cultural factors. By conducting the study in Peshawar, Pakistan, specific regional data on the prevalence and risk factors of anemia in IBD patients can be obtained. This would contribute to a better understanding of the local burden of anemia and aid in the development of region-specific management strategies.

Investigating the effectiveness and safety of treatments for anemia in IBD patients in Peshawar would shed light on the local treatment practices and outcomes. This would provide valuable insights into the current management approaches, including the use of iron replacement therapy, and help identify potential gaps or areas for improvement in the local healthcare system.

Socioeconomic factors can significantly influence the prevalence, risk factors, and outcomes of anemia in IBD patients. Conducting the study in Peshawar, Pakistan, would allow for an examination of the impact of socioeconomic factors specific to the region, such as access to healthcare, socioeconomic status, and nutritional patterns, on the occurrence and management of anemia in IBD patients. This would facilitate the development of targeted interventions to address socioeconomic disparities in anemia care.

Cultural beliefs, practices, and dietary habits can affect the prevalence and management of anemia in IBD patients. By conducting the research in Peshawar, Pakistan, the study can explore the cultural considerations related to anemia, such as dietary restrictions, traditional remedies, and adherence to treatment, which may impact treatment outcomes. Understanding these cultural factors can help tailor interventions and educational programs to improve patient acceptance and engagement with anemia management strategies.

Recommendations

Based on the findings of this study comparing treatments for anemia in patients with IBD, several recommendations can be made. Firstly, it is crucial to implement targeted iron replacement therapy for individuals with IDA in the context of IBD. This approach should consider the individual's disease activity, severity, and iron levels to ensure effective management while minimizing the risk of adverse events. Secondly, healthcare providers should carefully monitor patients receiving iron supplementation, particularly those with anemia of chronic disease, to prevent exacerbation of their condition. Close monitoring of hemoglobin levels, iron levels, and disease activity is essential to adjust treatment strategies accordingly. Additionally, healthcare professionals should educate patients about the importance of compliance with iron replacement therapy and the potential side effects that may arise. Lastly, further research is needed to explore alternative treatments and interventions that can address anemia in patients with IBD, taking into account the complex nature of the disease and its impact on iron metabolism. By implementing these recommendations, healthcare providers can enhance the management of anemia in patients with IBD and improve their overall well-being and quality of life.

## Conclusions

In conclusion, this study aimed to assess the effectiveness and safety of treatments for anemia in patients with IBD. The hypothesis that iron replacement therapy is effective in treating IDA in patients with IBD, but may exacerbate anemia of chronic disease in some cases, was investigated. The findings from the comparison of two groups, cases and controls, based on various variables, shed light on the differences and implications of anemia in the context of IBD. The study revealed that the case group, consisting of individuals with IBD, exhibited lower mean hemoglobin levels, lower iron levels, and higher disease activity and severity compared to the control group. These findings support the hypothesis that IDA is prevalent in IBD cases and can be effectively addressed through targeted iron supplementation. However, caution should be exercised as iron replacement therapy may worsen anemia of chronic disease in certain cases. Furthermore, the study highlighted the importance of considering potential adverse events associated with iron supplementation, particularly in cases of gastrointestinal side effects, allergic reactions, and iron overload. Overall, these findings contribute to the understanding of anemia management in patients with IBD, emphasizing the need for tailored treatment approaches to effectively address IDA while carefully considering individual patient characteristics and potential risks.
